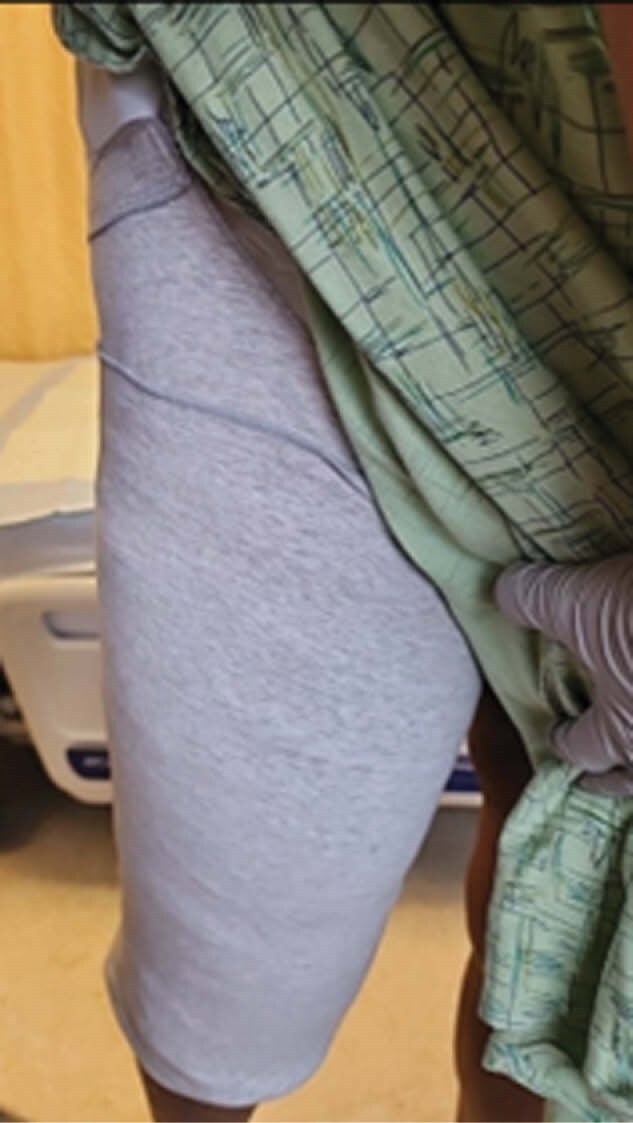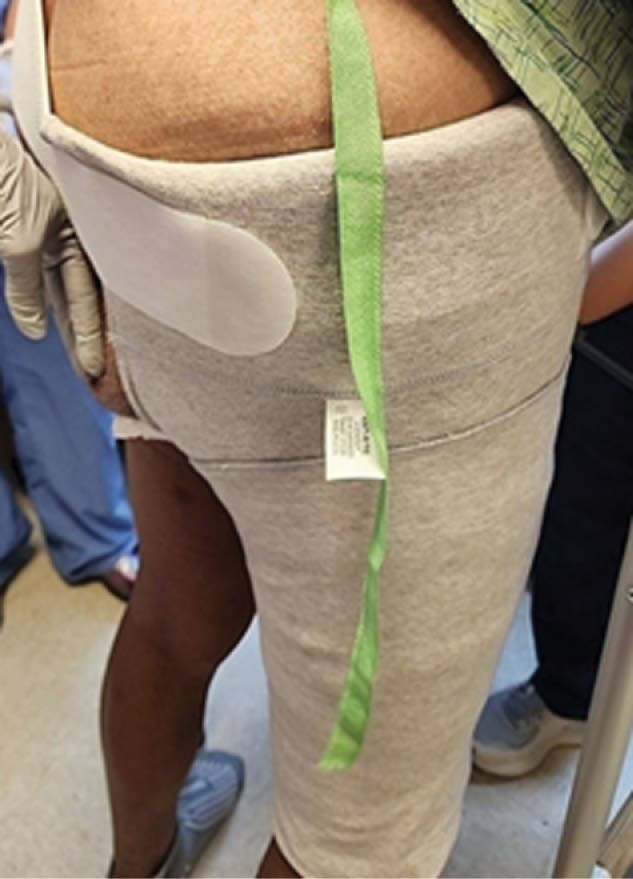# 1002 Novel Utilization of Rehabilitative Tool for Dressing Support and Compression in Periarea Skin Integrity Compromise

**DOI:** 10.1093/jbcr/iraf019.533

**Published:** 2025-04-01

**Authors:** Sara Twohey

**Affiliations:** Rhode Island Burn Center

## Abstract

**Introduction:**

Maintenance and positioning of groin and periarea dressings in burn and necrotizing soft tissue infection wound care present dressing integrity and functional mobility challenges for pts. and providers. Burn rounds discussion led to rehabilitative tool use to provide dressing support and compression w/improved functional capability.

**Methods:**

Case example of patient with necrotizing soft tissue infection in the inguinal area extending to perineum. Pt. underwent multiple surgical interventions over 9 wks: debridement of abdominal wall, groin, perineum and thigh; washout and negative-pressure application; allograft with failure requiring removal; flap autograft to groin from thigh donor w/negative-pressure application.

Evaluation noted deficits in range of motion, strength, functional mobility, and cognition. During functional tasks with therapy and nursing, dressing integrity and positioning was problematic due to location, drainage and care needs.

During rounds dressing integrity concerns were reviewed prior to discharge. Suggested use of above-knee amputation (AKA) shrinker sleeve for compression overlay made by physical therapy. The AKA shrinker sleeve can provide 25-30mmHg compression with dressing support and improvement in the patient’s functional care needs and independence. The shrinker sleeve was applied over primary dressing and ace wrap. The proximal sleeve edge supported the inguinal dressing w/the full sleeve length folded up for secondary compression.

**Results:**

The pt. was able to don the shrinker sleeve w/training to achieve functional progression during hospitalization before discharge to a rehabilitation facility. Compression support from the sleeve minimized migration of wound dressings. Dressing change frequency and reinforcement decreased w/benefits to faster wound healing, patient comfort and functional recovery.

**Conclusions:**

Use of an AKA shrinker sleeve for wounds at thigh and periarea minimized distal migration of dressings and need for reapplication. Pt. reported decreased pain w/dressing changes and improved functional independence. Use of the sleeve led to decreased dressing time requirements, increased efficiency and decreased need for dressing reinforcement.

**Applicability of Research to Practice:**

AKA shrinker sleeve use postop has proven successful in cases when dressings require added support. Use of the sleeve across the knee with custom stitching for contouring to patient’s limb has seen similar success. Decreased dressing time requirements may be associated with increased patient/caregiver satisfaction, projected to decreased LOS. Increased patient functional independence and healing w/decreased dressing compromise support the mission of the interdisciplinary team.

**Funding for the Study:**

N/A